# Supplementary figures and images for: Cost effectiveness of rituximab and mycophenolate mofetil for neuromyelitis optica spectrum disorder in Thailand: Economic evaluation and budget impact analysis
Source: PLoS One. 2020 Feb 12;15(2):e0229028. doi: 10.1371/journal.pone.0229028 (PMC7015451; doi:10.1371/journal.pone.0229028)

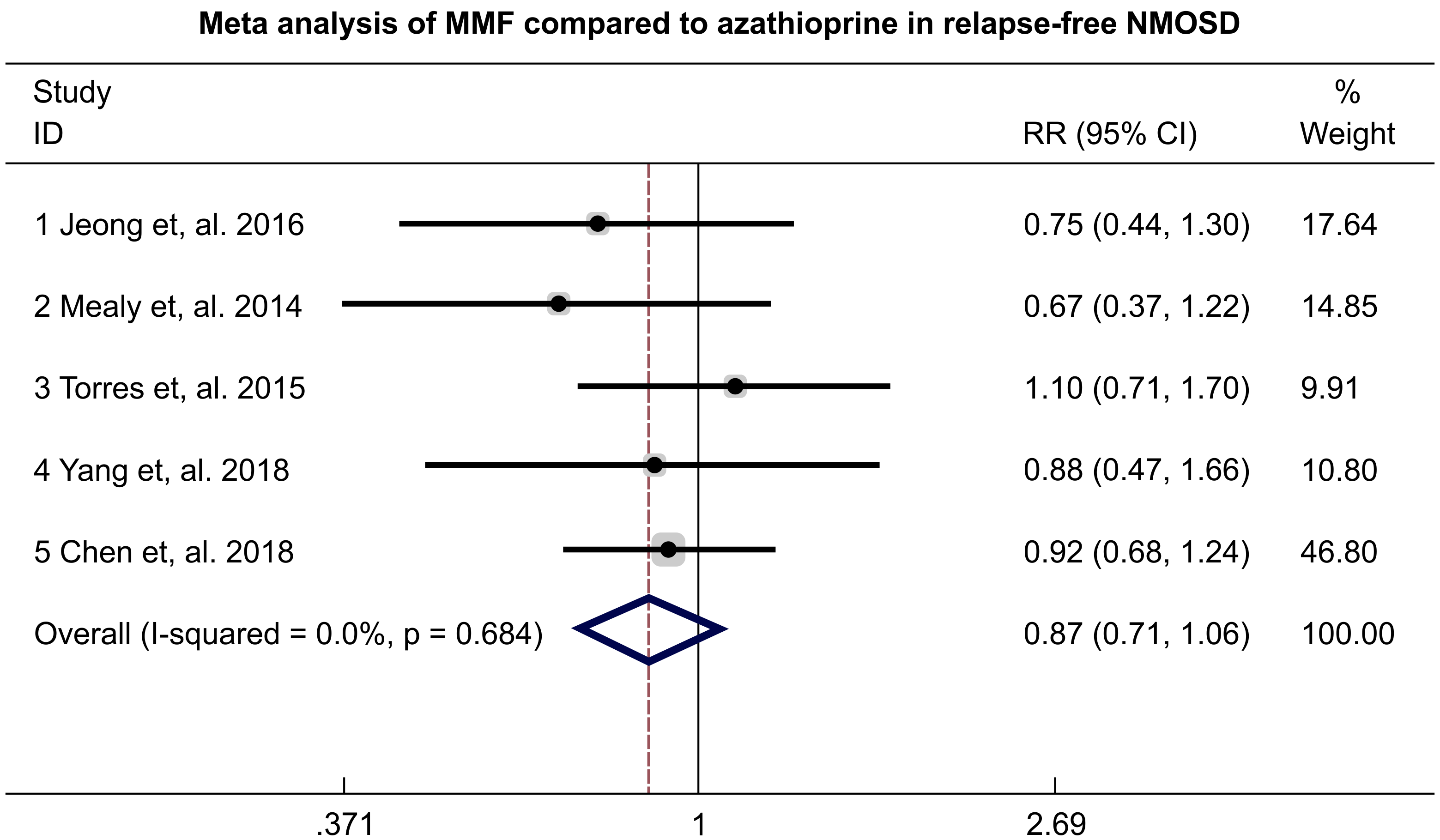

Supplement: S1 Fig — (TIF) [file pone.0229028.s001.tif]

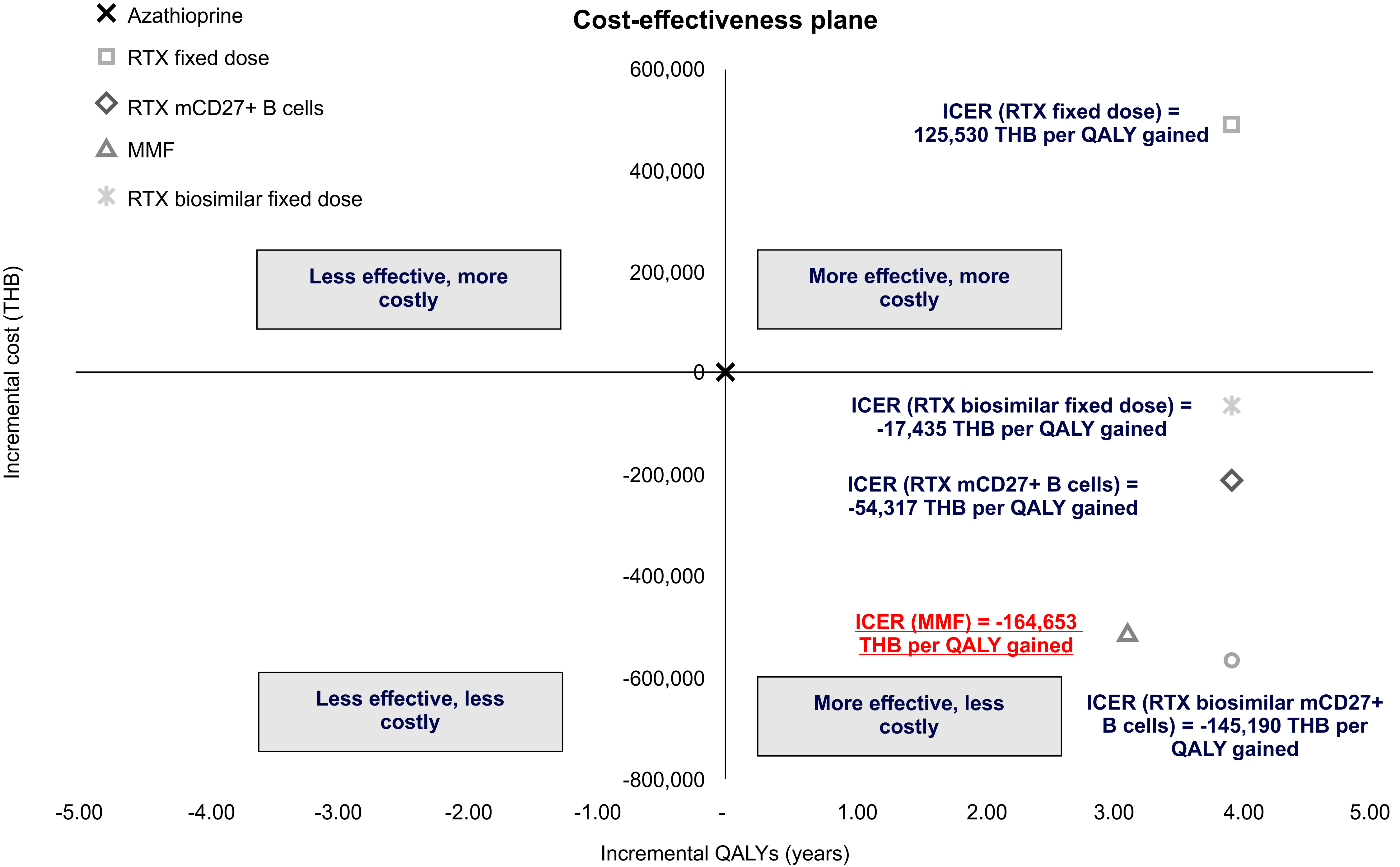

Supplement: S2 Fig — Cost-effectiveness plane covering lifetime cost effectiveness of six treatment options for NMOSD, after MMF price adjustment to 14.5 THB. RTX: rituximab; MMF: mycophenolate mofetil; mCD27+ B cells: monitor CD27+ B cells. (TIF) [file pone.0229028.s002.tif]

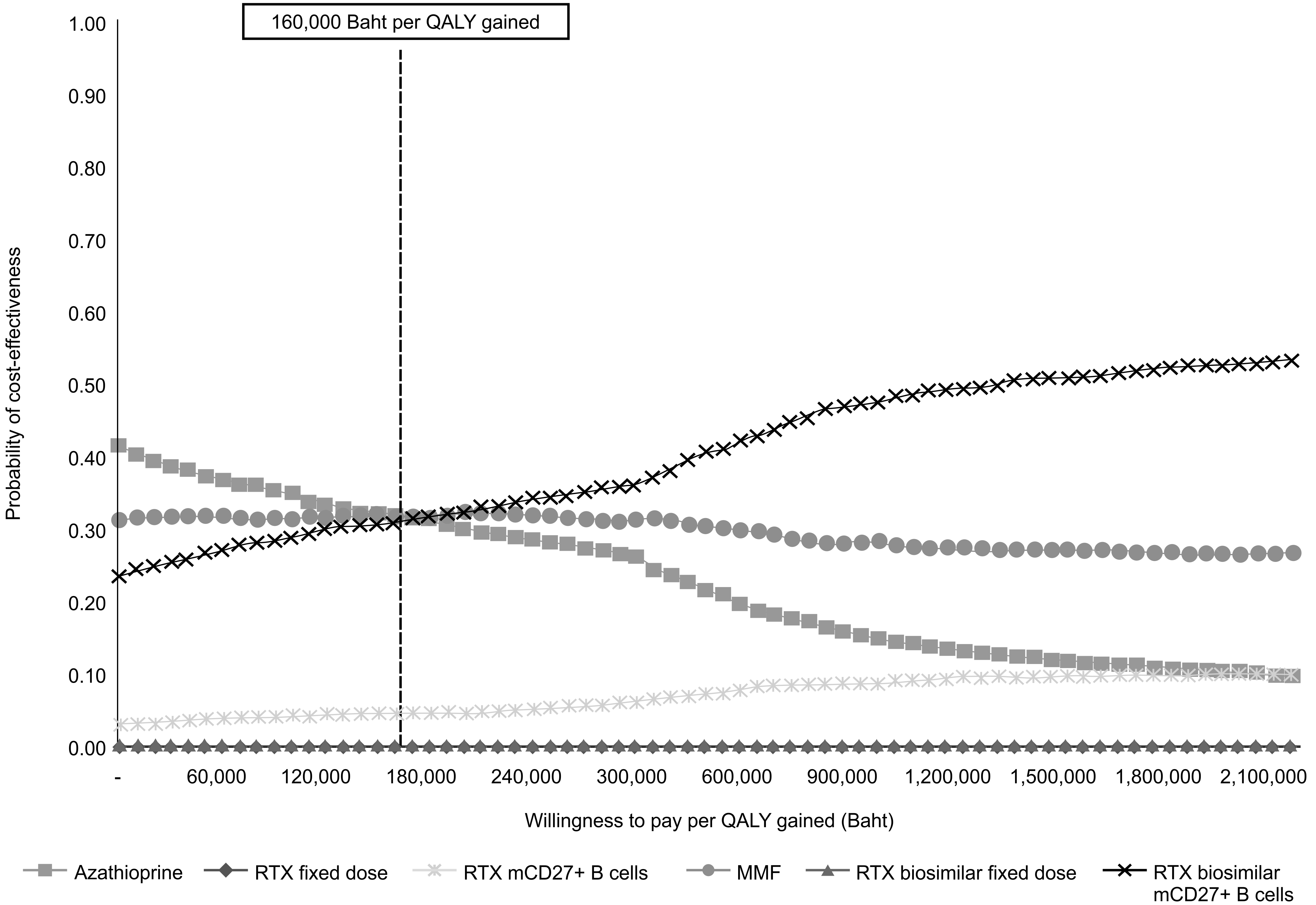

Supplement: S3 Fig — Acceptability curves of cost effectiveness at the different ceiling thresholds for six NMOSD treatment options, after the MMF price adjustment to 14.5 THB. RTX: rituximab; MMF: mycophenolate mofetil; mCD27+ B cells: monitor CD27+ B cells. (TIF) [file pone.0229028.s003.tif]
