# Supplementary material for: Cost effectiveness of rituximab and mycophenolate mofetil for neuromyelitis optica spectrum disorder in Thailand: Economic evaluation and budget impact analysis
Source: PLoS One. 2020 Feb 12;15(2):e0229028. doi: 10.1371/journal.pone.0229028 (PMC7015451; doi:10.1371/journal.pone.0229028)
Supplement: S1 Text — (DOCX) [file pone.0229028.s004.docx]

**Supporting Information**

**S1 text.** **Treatment efficacy of Mycophenolate mofetil**

Data on the efficacy of mycophenolate mofetil (MMF) versus azathioprine in randomized control trials are not available. We conduct the systematic review and meta-analysis with the aim of comparing the relapse free between Azathioprine and mycophenolate mofetil for treating Neuromyelitis optica spectrum disorder (NMOSD).

**Literature search**

The PUBMED and EMBASE databases was searched for articles on the efficacy of mycophenolate mofetil (MMF) in preventing NMOSD relapse, in comparison with azathioprine

**Population of interest**

Adults with neuromyelitis optica or NMOSD

**Treatment of regimens of interest**

This study compared the efficacy in preventing relapse, as well as the rate of relapse-free states, between MMF and azathioprine treatment regimens.

**Study selection**

Two reviewers independently evaluated titles and abstracts for selecting the studies. In cases when a definite decision could not be made based on the title and/or abstract alone, the full articles were retrieved for detailed assessment according to the inclusion criteria. Disagreements between the two reviewers were solved by consulting a third reviewer.

**Selection criteria**

Individual studies should meet all of the following inclusion criteria:

1. Clinical research with number of patients more than 10 patients
2. Studies published in English
3. Adult patients (aged 18 years or more)
4. The study compared the efficacy between mycophenolate mofetil and azathioprine
5. Outcome measures were relapse-free state and relapse reduction

**Flow chart of the study selection process**

38 studies identified

from Pubmed

63 studies identified

from Embase

13 studies deleted because of

duplication

88 studies were reviewed

by titles and abstracts

14 studies were reviewed

by full text

5

studies eligible for

review

823 studies excluded:

-

27

narrative reviews

-

14 non

-

therapeutic studies

-

16 case report

-

-

-

6 Non English article

-

-

-

1 guideline

2 systematic review

3 other disease group(MOG)

5 pedriatric group

9

studies excluded ;

-

4

duplicate

-

studies

-

5

no

comparator

**Outcomes**

The outcome of interest was relapse-free state under MMF and azathioprine treatment in NMOSD.

**Statistical analysis**

The meta-analysis was performed using STATA version 14. Risk ratios (RR) of relapse were estimated.
