# Supplementary material for: Cost effectiveness of rituximab and mycophenolate mofetil for neuromyelitis optica spectrum disorder in Thailand: Economic evaluation and budget impact analysis
Source: PLoS One. 2020 Feb 12;15(2):e0229028. doi: 10.1371/journal.pone.0229028 (PMC7015451; doi:10.1371/journal.pone.0229028)
Supplement: Attachment — Submitted filename: Rebuttal for Reviewer1 22-01-2020.docx [file pone.0229028.s005.docx]

Reviewer #1: Authors adequately replied to the points of the previous revision.

I have only few considerations (see below). Moreover, I highly recommend an accurate revision of English (I doubt a professional editing was performed, as, basically, no language corrections are present in the revised manuscript).

Major points:

1. Cost variables: Unfortunately, as 13% of patients are seronegative (and this population could have a different relapse-rate), Authors should at least add data regarding seronegative patients (do the ARR and disease severity differ from seropositive patients?). As the longitudinal population includes seropositive patients only, this should be taken into account in the statistical analysis or this point should at least be discussed.

Answer: We have added the demographic data of the seronegative patients to page 7 of the manuscript. Moreover, this point was added to the discussion section on page 18: “In other words, cost parameters generated by patient groups of mixed populations of seronegative NMOSD who exhibited lower relapse rates will lower the cost parameters more than expected. Those patients in the seropositive NMOSD group who more frequently relapse will incur higher costs for both direct and indirect medical expenses compared with the seronegative patients. However, if we apply only AQP4 positive NMOSD in the cost calculations, using high efficacy medication will result in greater differences in reducing relapse frequency and in further reducing the costs associated with treatments. Hence, the ICER that generates this situation negatively impacts our results, which indicates greater cost savings when treating NMOSD with high efficacy medications”.

Minor points:

1. THB and USD are not spelled out in the Abstract section.

This has been done.

2. Introduction: “NMOSD is a devastating CNS inflammatory demyelination”. Please, prefer “NMOSD is a devastating CNS inflammatory demyelinating disease”.

This has been done.

3. Material and methods: “Which began induction via intravenous rituximab [...]”. Please, revise the syntax of this sentence.

This sentence was revised to read, “which began with the introduction of two 1000 mg intravenous rituximab doses two weeks apart followed by 1000 mg intravenous rituximab doses every six months.”

4. Material and methods: USD is not spelled out. Moreover, I would move the exchange rate sentence to the beginning of the “Cost variables” section.

This has been done.

5. Clinical variables: I thank the Authors for adding the clinical and demographic features of the longitudinal NMOSD cohort, but please, revise the English.

This manuscript was sent to a new company (Elsevier language editing company) for English language editing after we completed the revisions. The certificate is attached to this revision.

6. Please, add USD to Table 2, Table 3 and page 16.

This has been done.

7. “The burden of NMOSD was recognized more than decade in Thailand”. Please, revise the English.

This sentence was revised as follows: “The issues with respect to NMOSD have been acknowledged for more than a decade in Thailand, i.e., since the NMO-IgG test had been made available…”

8. Discussion: “[…] inaccessible to high cost drugs. Unfortunately […]”. Please, consider revising (i.e., “[…] inaccessible to high cost drugs with consequent inadequate relapse prevention and disability accrual.”)

This has been done.

9. Discussion: “[…] which helped represent […]”. Consider revising (i.e. “[…] which helped representing […]”).

This has been done.

10. Please correct “these parameters was analysed” in “were analysed”.

This has been done.
